# Neuromuscular organisation and robustness of postural control in the presence of perturbations

**DOI:** 10.1038/s41598-019-47613-7

**Published:** 2019-08-22

**Authors:** Victor Munoz-Martel, Alessandro Santuz, Antonis Ekizos, Adamantios Arampatzis

**Affiliations:** 1 Department of Training and Movement Sciences, Humboldt-Universität zu Berlin, Berlin, Germany; 2 Berlin School of Movement Science, Humboldt-Universität zu Berlin, Berlin, Germany

**Keywords:** Central pattern generators, Computational neuroscience, Motor control

## Abstract

Perturbation-based exercise interventions challenge balance and improve reactive motor control. Our purpose was to investigate the modular organisation during a standing balance task in both stable and unstable conditions to provide new insights into the neuromuscular control mechanisms needed to cope with perturbations. Fifteen participants performed 54 cycles of a specific task (i.e. pass from a double- to a single-leg standing) on stable ground and an unstable oscillating platform (Posturomed). Muscle synergies were extracted from the electromyographic activity of thirteen lower limb muscles. The maximum Lyapunov exponents of different body segments were calculated using kinematic data. We found two synergies functionally associated with the single- and double-leg stance in both stable and unstable conditions. Nonetheless, in the unstable condition participants needed an extra muscle synergy also functionally related to the single stance. Although a simple organisation of the neuromuscular system was sufficient to maintain the postural control in both conditions, the increased challenge in the oscillating platform was solved by adding one extra synergy. The addition of a new synergy with complementary function highlighted an increased motor output’s robustness (i.e. ability to cope with errors) in the presence of perturbations.

## Introduction

For humans, maintaining balance is a necessary requirement not only during locomotion^1^ but in many other motor tasks as well^2–5^. Daily-life activities involve perturbations which challenge the neuromuscular system to modify its control strategies^6–8^. Challenging balance conditions and perturbations have been proposed as an effective exercise intervention to reduce fall risk in older adults^9–11^. Training programmes using unexpected or continuous perturbations to exercise the mechanisms of dynamic stability have the potential to enhance muscle strength as well as sensory information processing within the motor system^12^. Furthermore, perturbation-based interventions improve reactive balance control in post-stroke^13,14^ and Parkinson’s disease patients^15^. The reaction to a perturbation is related to the type of perturbation, whilst a large perturbation may require a recovery movement a small perturbation will not necesarlly modify the motor behaviour^16^. Both abilities, cooping with large and small perturbations are key components for a stable motor output^17^. The sensitivity of any system to small perturbations is normally referred as “local stability”^18^ and is crucial for the execution of a task uninterruptedly in dynamic conditions^19,20^. The maximum Lyapunov exponent (MLE) is a measure of the local dynamic stability and is considered to reflect the ability of dynamical systems -such as humans during gait- to withstand perturbations^17,18,21,22^. The theoretical concept of the MLE suggests that although the entire dynamic of the system can be approximated by measuring only one site^23,24^, assessing different components of the system may also provide specific information about the sub-system being evaluated^21^.

There is little information about how muscle activations are organized to control the body in the presence of perturbations. Nonetheless, challenging motor control strategies through perturbations is an effective way to investigate the neuromuscular responses to unstable conditions^7^ and could highlight possible neuromuscular mechanisms responsible for the positive effects of perturbation-based interventions.

A generally accepted idea is that the central nervous system (CNS) might simplify the production of movement by activating muscles in common patterns called synergies^25–27^. Instead of activating each muscle individually, the CNS might create a motor output by combining small sets of time-dependent commands (motor primitives) and time-independent weights (motor modules) that create patterns in muscles^26–28^. It has been proposed that synergies may be specific to each task^29^. This task-related control could allow for fast reconfigurations when the task demands change^30,31^. During walking and running, although the general modular organisation remains unaltered in the presence of perturbations, a modification of the temporal components of the muscle synergies, characterized by a widening of the motor primitives, has been reported^7,32^. This widening increases the overlap of chronologically adjacent synergies and has been interpreted as a motor control strategy that is used to increase the robustness of the neuromuscular system’s output while performing a task^7,16,33^. Kitano proposed that a biological system is evolutionally robust when its characteristics can withstand perturbations or uncertainty^34^. In a similar manner, robustness can be defined as the ability of the CNS to cope with perturbations or with errors of execution^7^. Therefore, using perturbations offers advantages to study the neuromuscular responses that might be providing robustness to the neuromuscular system’s output, and be, consequently, related to the effectiveness of fall prevention programs. Hence, the purpose of the current study was to investigate the modular organisation in healthy young adults during a standing balance task on a stable and an unstable platform in order to improve our understanding of the neuromuscular control mechanisms in the presence of external perturbations. We hypothesized an increased robustness of the motor output in the unstable compared to the stable condition, achieved through a reorganisation of the time-dependent activation coefficients (motor primitives) of muscle synergies.

## Methods

### Experimental protocol

We recruited 15 healthy adults (11 males, 4 females, height 1.75 ± 0.10 m, body mass 67 ± 11 kg, age 28 ± 5 years). The sample size was a priori calculated based on the aforementioned motor primitive’s modification in the presence of perturbation during locomotion^7^. All participants were regularly active and had no history of neuromuscular or musculoskeletal impairments, nor any injury at the time of the measurements or in the previous six months. The Ethics Committee of the Humboldt-Universität zu Berlin reviewed and approved the study design (HU-KSBF-EK_2018_0013). All the participants gave written informed consent for the experimental procedure, in accordance with the Declaration of Helsinki. Kinematics data were recorded through a ten infrared-camera motion capture system (Vicon, Oxford, U.K.) operating at 250 Hz. The activity of 13 ipsilateral muscles was recorded using a 16-channel wireless electromyography (EMG) system (Myon m320, Myon AG, Schwarzenberg, Switzerland), with a sampling frequency of 1 kHz.

**Figure 1:**
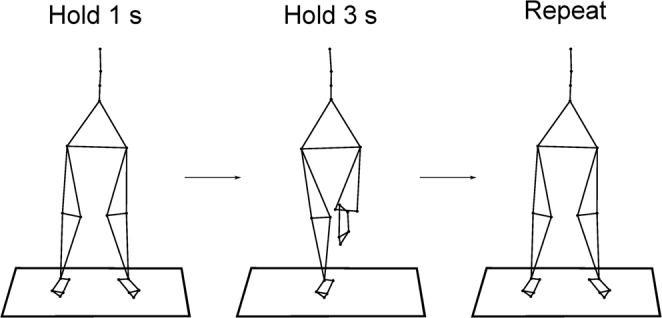
Description of the performed task. Participants were asked to pass from a double- to a single-leg stance, maintain the position for 3 s, return to the bipedal position and after 1 s repeat the task.

**Figure 2:**
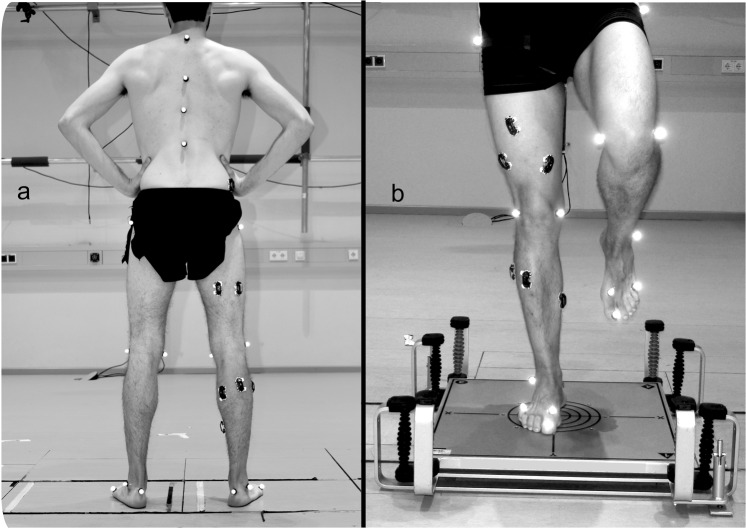
Reflective markers and EMG sensors position. Panel “a” shows the hard ground condition and panel “b” shows the damped oscillating platform used as unstable ground condition.

The participants were asked to pass from an initial double- to a single-leg standing on the right foot (DLS and SLS, respectively), maintain the SLS position for 3 s and return to the DLS. The whole cycle, defined as the time between two consecutive foot lift-offs, lasted for 6 s and the task was then immediately repeated (Fig. 1). A metronome aided with timing the task. The participants performed 54 cycles, on two different surfaces: hard uniform stable ground (SG) and damped oscillating unstable platform (Unstable Ground – UG, Posturomed Haider GmbH, Germany). The platform consisted of a 60 * 60 cm plate suspended by a double swinging mechanism that responded to any force application with a maximum damped displacement of 50 mm to the sides and 80 mm in the anteroposterior direction with an oscillation frequency between 1.0 and 3.2 Hz. (Fig. 2). The order of conditions was randomized.

### Cycle assessment

Sixteen reflective markers were placed bilaterally on the following anatomic landmarks: greater trochanter, lateral and medial epicondyle of the femur, Achilles tendon insertion on the calcaneus, lateral malleolus, tip of the first toe, dorsal margin of the fifth and first metatarsal heads. The second, seventh and tenth thoracic and the second lumbar *vertebrae* were marked as well. The cycle breakdown was obtained from the kinematics of the foot (calcaneus, toe tip, fifth and first metatarsal). This data was low-pass filtered using a 4^th^ order IIR Butterworth zero-phase filter with cut-off frequency of 50 Hz^35^. Touchdown was estimated using the modified foot contact algorithm developed by Maiwald *et al*.^7,35^. For assessing lift-off, we used the foot acceleration and jerk algorithm^7^. The algorithm searches for the vertical acceleration’s global maximum of the fifth metatarsal between two consecutive touchdown events to estimate the lift-off (LOe, where the “e” stays for “estimated”). To get closer to the “real” lift-off timing, a characteristic minimum in the vertical acceleration (i.e. when the jerk equals zero) of the fifth metatarsal marker is identified in a reasonably small neighbourhood of the LOe. We found [LOe − 50 ms, LOe + 200 ms] to be the sufficiently narrow intervals needed to make the initial lift-off estimation. Since all participants, in the SG condition, stepped with the left foot onto a force plate (AMTI BP600, Advanced Mechanical Technology, Inc., Watertown, MA, USA) we assessed the performance of both approaches in this condition against true values assessed from kinetic data. True errors were of 9 ± 6 ms for the estimation of touchdown and 13 ± 9 ms for the estimation of lift-off. To avoid inaccuracies, the first and last two cycles were removed from each data set and the central 50 cycles were kept for further analysis.

### EMG recording and processing

The activity of the following 13 ipsilateral (right side) muscles was recorded: *gluteus medius (ME)*, *gluteus maximus (MA)*, *tensor fasciae latae (FL)*, *rectus femoris (RF)*, *vastus medialis (VM)*, *vastus lateralis (VL)*, *semitendinosus (ST)*, *biceps femoris (long head*, *BF)*, *tibialis anterior (TA)*, *peroneus longus (PL)*, *gastrocnemius medialis (GM)*, *gastrocnemius lateralis (GL)* and *soleus (SO)*. The EMG signals were high-pass filtered and then full-wave rectified and low-pass filtered using a 4^th^ order IIR Butterworth zero-phase filter with cut-off frequencies of 50 Hz (high-pass) and 20 Hz (low-pass), respectively^7,36^ using R v3.4.4 (R Found. for Stat. Comp.). After subtracting the minimum, the amplitude was normalised to the maximum activation recorded in each trial^37^. Each cycle was time-normalised to a length of 1200 points through resampling the data. To approximately maintain the ratio between the SLS and DLS timing, we assigned 800 points to the SLS and 400 points to the DLS.

### Muscle synergies assessment

The classical Gaussian non-negative matrix factorisation (NMF) algorithm^7,38^ was used for the extraction of muscle synergies from EMG data through a custom script^39^ (R v3.4.4, R Found. for Stat. Comp.). The time-dependent muscle activity vectors were grouped in an *m* × *n* matrix *V*, where *m* = 13 (number of muscles) and *n* = number of normalised time points. This matrix was factorised such that *V ≈ V*_*R*_ = *WH*. The new reconstructed matrix *V*_*R*_ approximates the original matrix *V*. *H* represents the motor primitives matrix^36,40^ containing the time-dependent coefficients of the factorisation with dimensions *r* × *n*, where *r* represents the number of synergies necessary to sufficiently reconstruct the EMG signals. The *m* × *r* motor modules matrix *W*^36,41^, contained the time-invariant muscle weightings. *H* and *W* described the synergies necessary to accomplish a movement. The update rules for *H* and *W* are presented in Eqs 1.1 and 1.2.

1.1 $${H}_{i+1}={H}_{i}\frac{{W}_{i}^{T}V}{{W}_{i}^{T}{W}_{i}{H}_{i}}$$

1.2 $${W}_{i+1}={W}_{i}\frac{V{({H}_{i+1})}^{T}}{{W}_{i}{H}_{i+1}{({H}_{i+1})}^{T}}$$

The limit of convergence was reached when a change in the calculated *R*^2^ between *V* and *V*_*R*_ was smaller than the 0.01% in the last 20 iterations^7,36,42^, meaning that, with that amount of synergies, the signal could not be reconstructed any better. This operation was first completed by setting the number of synergies to 1. Then, it was repeated by increasing the number of synergies each time, until a maximum of 10 synergies. The number 10 was chosen to be lower than the number of muscles, since extracting a number of synergies equal to the number of measured EMG activities would not reduce the dimensionality of the data. Specifically, 10 is the rounded 75% of 13, which is the number of considered muscles. The computation was repeated 10 times for each synergy, each time creating new randomised initial matrices *H* and *W*, in order to avoid local minima^7,43^. The solution with the highest R^2^ was then selected for each of the 10 synergies.

The minimum number of synergies required to represent the original signals was chosen fitting the curve of *R*^2^ values versus synergies using a simple linear regression model for all the synergies. The mean squared error was then repeatedly calculated, each time removing the lower synergy point, until only two points were left or until the mean squared error fell below 10^−5^ ^7,36^. The extracted synergies were classified based on the timing of motor primitives’ global maxima. Following previous definitions^7,36^ only fundamental primitives (i.e. showing a single activation peak) were considered. When two or more fundamental synergies are blended into one, a combined synergy appears. Combined synergies usually constitute, in our data, 10 to 20% of the total extracted synergies. Due to the lack of consent in the literature on how to interpret them, we excluded the combined synergies from the analysis.

### Metrics for comparison of curves

In order to compare the motor primitives of both conditions, we evaluated the centre of activity (*CoA*) and full width at half maximum (FWHM). The *CoA* was defined as the angle of the vector (in polar coordinates) that points to the centre of mass of that circular distribution^7,44^. The polar direction represented the cycle’s phase, with angle 0 ≤ *θ*_*t*_ ≤ 2π. The following equations define the *CoA*:

2.1 $$A=\mathop{\sum }\limits_{t=1}^{p}(\cos \,{\theta }_{t}\times {P}_{t})$$

2.2 $$B=\mathop{\sum }\limits_{t=1}^{p}(\sin \,{\theta }_{t}\times {P}_{t})$$

2.3 $$CoA=\arctan (B/A)$$

where *p* is the number of points of each cycle (*p* = 1200) and *P* is the activation vector. The FWHM was calculated as the number of points exceeding each cycle’s half maximum, after subtracting the cycle’s minimum^7,44^.

### Local dynamic stability assessment

We calculated the point-by-point Euclidean norm of the vectors containing the 3D-coordinates of the reflective markers, thus converting the three components (xi, yi, zi) to a single value ${n}_{2}=\sqrt{{x}_{i}^{2}+{y}_{i}^{2}+{z}_{i}^{2}}$. The resulting data was filtered with a 4th order IIR Butterworth zero-phase filter with a low-pass cut-off frequency of 20 Hz. The anatomical regions of interest were then represented by the respective markers: spine (the 2nd, 7th, 10th thoracic and 2nd lumbar vertebrae), pelvis (greater trochanter), knee (lateral and medial epicondyle of the femur) and foot (lateral malleolus). When two or more markers were related to a region (i.e. spine and knee), a point-by-point average was calculated for each marker group after filtering. The resulting single-vector time series for each right lower limb’s region and spine were used for further analysis and calculation of the Maximum Lyapunov Exponent. To avoid dependencies, we used the maximum number of shared cycles (50) for all trials and participants^21,45^, and excluded the first and last cycle (analysing a total of 48 cycles per participant), one participant was excluded from the analysis due to missing data. The coordinates of the data segments corresponding to the exact number of cycles were then extracted and normalised to a uniform length. The high number of analysed cycles ensured the reliability of the measurements based on our previous studies on locomotion^21,46^. Moreover during our pilot tests we noticed that after the designated number of repetitions fatigue began to set in.

State space reconstruction was achieved through delay coordinate embedding^47,48^, for each point of the time series and its time-delayed copies as follows:

3.1 $$S(t)=[z(t),z(t+\tau ),\,\ldots ,\,z(t+(m-1)\tau )]$$

with *S(t)* being the *m*-dimensional reconstructed state vector, *z(t)* the input 1D coordinate series, *τ* the time delay and *m* the embedding dimension. Time delays were calculated for each time series from the first minimum of the mutual-information curve, based on the Average Mutual Information function^49^.

Different values of *τ* and *m* can yield very different state-space reconstructions^50–52^. It is therefore suggested that optimised values of *τ* and *m* are necessary to best represent a dynamical system^21,53^. In the current study dimension of 3 was sufficient^21,54^ and time delays were individually chosen for each participant and each analysed segment^53^. Time delays were approximately 0.33 of the cycle length which is common in human movement studies^21,54^. Following the reconstruction of the times series, the Rosenstein algorithm was used to compute the average exponential rate of divergence of the trajectories in the state space, by calculating the linear distance of each point’s trajectory divergence to its closest trajectory^18,23^. The MLE were then calculated from the slope of the linear fit in the resulting divergence curves from 0 to 0.25 of a whole cycle. Analysis of the data was performed on MATLAB 2014b (Mathworks Inc., Natick, United States). Higher values in MLE indicate increased instability of the system.

### Statistics

To compare *CoA* and FWHM, we used a two-way analysis of variance (ANOVA) with repeated measures, using standing synergy (SLS, DLS) and condition (SG, UG) as within-subjects factor followed by a Tukey *post-hoc* analysis with false discovery rate *p-value* adjustment. To compare modules between conditions we adopted the same procedure using muscle (number of muscles) and condition as within-subjects factor. A two-way ANOVA for repeated measures was performed with anatomical region (spine, pelvis, knee, foot) and condition (SG, UG) as within-subjects factor on the MLE. A Bonferroni-corrected *post-hoc* analysis was conducted in the case of a significant time effect or interaction of the factors anatomical region and condition. All the significance levels were set to α = 0.05 and analyses were conducted on R v3.4.4.

## Results

### Cycle parameters

The duration of the cycles (lift-off to lift-off) did not differ when switching from SG to UG (6.035 ± 0.109 s and 6.013 ± 0.073 s for SG and UG, respectively, p = 0.522). The average duration of the SLS did not show differences between conditions either (stable = 3.684 ± 0.509 s, unstable = 3.512 ± 0.534 s, p = 0.374). Nonetheless, participants in the unstable condition showed an increased variability expressed in a significant larger variance (stable = 0.137 ± 0.069 s, unstable = 0.307 ± 0.270 s, paired t-test p = 0.012).

### Local dynamic stability

The MLE was significantly higher at the ankle compared to proximal segments (F (3,39) = 15.909, p = 0.001, η^2^ = 0.550) regardless the ground condition. Furthermore, there was an interaction between anatomical region and condition group (F (3,39) = 6.866, p = 0.02, η^2^ = 0.346). The MLE was significantly lower at the spine on the unstable ground (p = 0.006, 95% C.I = 86.7:97.7 for the SG and 75.7:86.6 for UG, Table 1). There were no differences for the pelvis (p = 0.444) nor the knee (p = 0.754) or foot among conditions (Table 1).

**Table 1:** Maximum Lyapunov exponent between conditions (stable and unstable ground) for every analysed anatomical region. The values are presented in mean ± standard deviation, positive differences (Δ > 0) denote higher values in the unstable condition. Asterisks denote statistically significant (p < 0.05) differences. Post hoc analysis are Bonferroni corrected.

Maximum Lyapunov Exponent					
Region	F (3,13) = 15.909, p = 0.001*, η^2^ = 0.550				
Post hoc		Mean ± sd	Δ	p. value	Effect size
Foot (150.5 ± 16.5) compared to	Knee	88.4 ± 4.4	61.7 ± 13.3	0.003*	0.96
	Pelvis	87.9 ± 2.9	62.5 ± 16.0	0.011*	0.98
	Spine	86.7 ± 1.8	63.7 ± 16.4	0.011*	0.99
**Condition**	**F(1,13)** = **0.018, p** = **0.895, η2** = **0.001**				
**Interaction (Region by Condition)**	**F(3,39)** = **6.866**, p = **0.002**, **η2** = **0.346**				
**Post-hoc**	**Stable**	**Unstable**	**Δ**	***p. value***	**Effect size**
Foot	142.7 ± 54.0	158.4 ± 70.7	11.0%	0.030	0.65
Knee joint	89.3 ± 18.6	88.2 ± 17.4	−1.3%	0.754	−0.08
Pelvis	89.2 ± 11.2	86.8 ± 13.5	−2.7%	0.444	−0.21
Spine	92.2 ± 9.5	81.2 ± 9.4	−11.9%	0.006*	0.86

### Modular organisation

**Figure 3:**
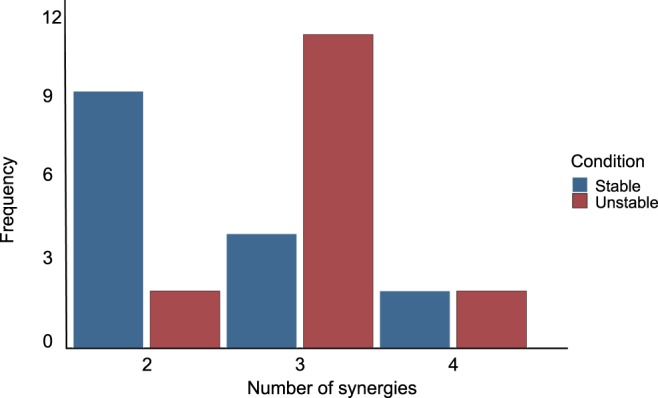
Frequency distribution of the minimum number of synergies necessary to sufficiently reconstruct the EMG signals recorded from all participants on stable and unstable ground. Significant differences were observed for the mean (2.5 ± 0.7 for the stable and 3.2 ± 0.5 for the unstable condition, p = 0.029) and median values (2 for the stable and 3 for the unstable condition, p = 0.035).

**Figure 4:**
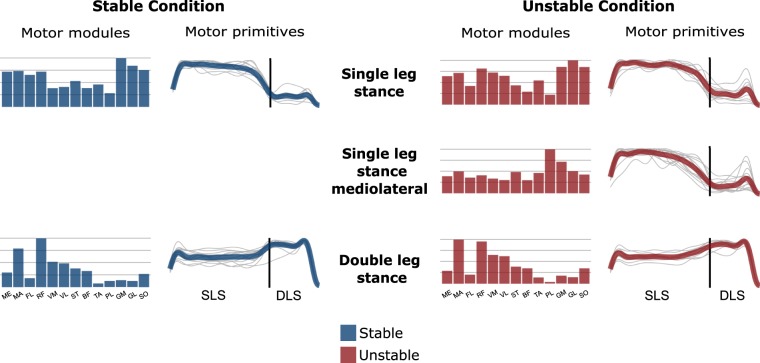
Average motor modules and motor primitives of the fundamental synergies needed to perform the postural task on stable and unstable ground. The motor modules are presented on a normalised y-axis base. For the motor primitives, the x-axis full scale represents one cycle (lift-off to lift-off, time-normalised to the same amount of points, the vertical line indicates the touchdown, i.e. the beginning of the double leg stance) and the y-axis the normalised amplitude. SLS = single leg stance, DSL = double leg stance, ME = gluteus medius, MA = gluteus maximus, FL = tensor fasciae latae, RF = rectus femoris, VM = vastus medialis, VL = vastus lateralis, ST = semitendinosus, BF = biceps femoris (long head), TA = tibialis anterior, PL = peroneus longus, GM = gastrocnemius medialis, GL = gastrocnemius lateralis and SO = soleus.

For all the trials, a minimum of two synergies and a maximum of four were sufficient to satisfactorily reconstruct the measured EMG activity (median = 2 and 3 for SG and UG respectively). More synergies were needed to reconstruct the trials of the unstable (mean = 3.2 ± 0.5) compared to the stable condition (mean 2.5 ± 0.7, paired t-test p = 0.029, Fig. 3). In both conditions, the fundamental activation patterns were associated with temporally different phases of the task (Fig. 4). The first synergy was shared between conditions and functionally referred to the SLS (peak at ~9% and ~16% of the cycle for stable and unstable, respectively) and showed a major involvement of hip abductors, hip extensors and plantar flexors. The second synergy, which was also shared among conditions, described the DLS (peak at ~90% and ~89% of the cycle for stable and unstable, respectively) and showed a main contribution of knee extensors and flexors and *hip extensors*. The extra synergy for the unstable ground condition was functionally related to the SLS (peak at ~21%) and was mainly characterized by the involvement of mediolateral stabilizers of the lower leg. For this reason, from now on it will be referred to as SLS mediolateral synergy. Since this synergy was only present in the UG condition, comparisons between SG and UG conditions were performed for the SLS and DLS synergies exclusively.

There were no differences for the shared motor primitives of the SLS and DLS in either the FWHM or the CoA between SG and UG (F (1,14) = 8.16, p = 0.201, Table 2). Similarly, the motor modules in any of the shared synergies (SLS and DLS) did not differ between conditions (F (12, 364) = 0.28, p = 0.972 for the SLS and p = 0.267 for the DLS).

**Table 2:** Differences for motor modules and primitives between ground conditions. Motor primitives are compared by means of full width at half maximum (FWHM) and centre of activity (CoA). Standing (SLS) and double leg standing (DLS) synergies were shared by both the stable and unstable ground condition, while a new synergy (SLS mediolateral) was found only in the unstable trials. For this reason, we only presented the comparison between stable and unstable, where positive differences (Δ > 0) denote bigger values in the unstable condition, whereas negative differences imply lower values.

Motor Primitives	FWHM		Effect size	CoA		Effect size
	Δ	p-value		Δ	p-value	
SLS stable vs. SLS unstable	−0.4%	0.977	−0.10	+ 2.3%	0.344	0.32
DLS stable vs. DLS unstable	− 6.2%	0.440	−0.22	−2.1%	0.577	0.14
**Motor Modules**	**p-value**					
SLS stable vs. SLS unstable	0.972					0.15
DLS stable vs. DLS unstable	0.267					0.22

## Discussion

In the present study, we investigated the modular organisation of a standing balance task on stable and unstable ground in order to improve our understanding of the neuromuscular control mechanisms adopted by the CNS to maintain motor task functionality during external perturbations. Our results show that a very simple organisation of the neuromuscular system is sufficient to maintain the postural control in DLS and SLS on both SG and UG. In the SG condition, two synergies were sufficient to describe the modular organisation of the task, one for each stance, and achieve the functional goal of keeping the upright posture. In the UG condition, the increased challenge of postural stability was solved by adding one extra synergy during the SLS.

Stability increased (lower MLE) from the distal (foot) to the proximal (spine) anatomical regions in both SG and UG. Remarkably, this phenomenon was more pronounced in the unstable condition. The lower MLE from the spine in UG compared to SG suggest that the neuromuscular system increased the stability of the trunk in relation to the foot to a higher extent in presence of distal perturbations. Previous studies reported a stability prioritization of proximal over distal segments during balancing and walking^55–58^. Our results show that this phenomenon (i.e. priority of proximal segment stability) is facilitated in the perturbed condition. It has been shown that stability of the head is critical to obtain visual and vestibular references that are crucial for dynamic postural control^59–61^. In balance-challenging conditions, the integration of visual and vestibular information for effective postural control may be more relevant than in less challenging tasks, thus requiring higher trunk-head stability. Furthermore, our data indicate that the preservation of the task functionality in the presence of perturbations was achieved at the expense of accuracy: the variability of the cycle duration was twice as high (p = 0.012) in UG compared to SG.

It is well known that muscle activity is organized to control the displacement of the centre of mass by controlling the centre of pressure during upright posture^58,62,63^. The SLS synergy modules showed a main contribution of ankle (PL, GM, GL and SO) and hip muscles (MA, FL, ME), whilst in the DLS synergy, the main contribution was provided from *rectus femoris* and *gluteus maximus*. These two synergies remained unaffected by the change of ground condition (stable or unstable) in their spatial (i.e. motor modules) and their temporal (i.e. motor primitives) structure. In the UG condition, the displacement of the base of support amplified the need to compromise between keeping balance and maintaining the upright posture^2^. For this reason, any attempt to control the centre of mass necessarily results in a displacement of the base of support. From a mechanical point of view, these reciprocal constraints change the behaviour of the body from an inverted pendulum to a balancing pole^64,65^. As stated above, the incremented postural stability challenge was solved by adding one extra synergy during the SLS. This new synergy was present in most of the participants (73.3%) and was characterised by a dominant contribution of the shank muscles, especially the *peroneus longus*. It has been reported that distal muscles are more sensitive to perturbations than proximal muscles^66^. This could be due to specific morphological and anatomical properties (i.e. short fascicles, long tendons, and large pennation angles) that allow these muscles to be particularly sensitive to perturbations happening at low levels of force^67^.

Based on previous results from our group, we expected a conservation of the modular organisation of the system (i.e. same number of synergies) with a modification of its time coefficients (widening of motor primitives) leading to an increased motor output’s robustness^7^. A “robust adaptation” in response to perturbations is observed when (a) the state of the system is modified and the system is able to return to its original attractor or (b) the system moves to a new attractor that is able to respond adequately to perturbations maintaining its functionality^34^. The ability to maintain specific functionalities by changing the modes of operation in a flexible way is a characteristic of robust adaptation^34^. Considering the observed addition in the number of synergies as a modification of the state of the system and the fact that all participants managed to perform the task in face of perturbations, we can assume that functionality was maintained, despite an alteration of the modular organisation when comparing SG and UG tasks. Modularity is often presented as a biological design principle that allows robust responses^34,68^. Muscles synergies represent neural sets of task-specific modules that can be selected and combined for the production of different movement patterns^69,70^. The performed task was partially mechanically constrained by maintaining the upright standing position on one and two legs and on stable and unstable ground. Considering that a task-specific mechanical goal is likely to be reflected in a task-specific muscle synergy^43,71^, our results support the idea that for the induced perturbations, the control system increased its robustness by adding a new synergy with different muscle organisation, but complementary target function. In other words, while the shared SLS synergy is likely responsible for keeping the upright posture, the added synergy might be responsible for controlling the perturbations imposed by the displacement of the base of support. During the DLS, despite the presence of the same kind of perturbations (i.e moving ground), there was no necessity for an extra synergy. This might be due to the bigger base of support that provided larger boundaries of stability^60,72,73^.

Recent studies reported that perturbation-based training programmes using continuously variable and partly unpredictable disturbances can improve the neuromuscular control of the motor system and increase its stability during sudden balance recovering tasks^12,74^. Furthermore, it has been proposed that exercise including small continuous and unpredictable perturbations may introduce a more robust response to large perturbations by improving the modular organisation of the control system^16^. In highly challenging conditions, humans increase the fuzziness of the temporal boundaries in the modular organisation of walking and running and create a “buffer” of motor control enhancing the robustness needed to cope with external perturbations^7^. In this notion and considering our results, we interpret the addition of a new independent synergy as a “safety buffer” created by the neuromuscular system to minimize the effects of perturbations on the motor output.

Feedback-based control is crucial for robust locomotion^75^ and one of the main balance recovery mechanisms when perturbations are large or unexpected^76^. During bipedal balance tasks, in which distal segments are the first to move after a perturbation, proprioceptive pathways provide extremely fast feedback information^77^. However, large corrective responses undergo bigger time delays before being detectable^65,77^. These delays might be overcome by adaptive control strategies able to make up for the temporary lack of feedback^65,78^. Given the fundamental role of proprioception for feedback-based responses^33,77,79^, we reasoned that the additional synergy, mainly involving lower leg muscles, could promote the adaptive control of posture. This might happen by allowing the control of the base of support after perturbation with the smallest possible latency^77^.

Our results support the idea that the CNS takes advantage of sensorimotor integration to ensure robust control^65,80^ and that a modular organisation facilitates robustness^7,34^. Furthermore, the increased control’s robustness in the presence of external perturbations might be one important neural mechanism contributing to stability performance and could be of special interest for training and rehabilitation designs. For the latter, the aforementioned sensitivity of lower leg muscles to perturbations might explain why perturbation-based training programmes promote strength increase in these muscles^12^. However, perturbations must be challenging enough to engage or trigger the additional response to having a training effect^12^.

In conclusion, our results support the idea that the addition of a new synergy was a strategy to increase the robustness (i.e. ability to cope with errors) of the system’s motor output to perturbations. The new synergy was characterised by a major contribution of the lower leg muscles and had a temporal profile that was similar to the one of the SLS synergy. Such temporally co-existing synergies are likely to have different but complementary goals, in this case keeping the upright posture and controlling the displacement of the base of support. Moreover, modularity in the neuromuscular system might be an important feature to ensure robustness by providing a source to adaptive control strategies depending on the task characteristics.

## Data Availability

The datasets generated and analysed during the current study are available from the corresponding author on reasonable request.
